# Using Airborne Hyperspectral Imaging Spectroscopy to Accurately Monitor Invasive and Expansive Herb Plants: Limitations and Requirements of the Method

**DOI:** 10.3390/s19132871

**Published:** 2019-06-28

**Authors:** Dominik Kopeć, Agata Zakrzewska, Anna Halladin-Dąbrowska, Justyna Wylazłowska, Adam Kania, Jan Niedzielko

**Affiliations:** 1 Department of Geobotany and Plant Ecology, Faculty of Biology and Environmental Protection, University of Lodz, 90-237 Łódź, Poland; 2 Definity Sp. z o.o., 52-116 Wrocław, Poland; 3 MGGP Aero Sp. z o.o., 33-100 Tarnów, Poland

**Keywords:** invasive species, airborne, hyperspectral, sampling designs

## Abstract

Remote sensing (RS) is currently regarded as one of the standard tools used for mapping invasive and expansive plants for scientific purposes and it is increasingly widely used in nature conservation management. The applicability of RS methods is determined by its limitations and requirements. One of the most important limitations is the species percentage cover at which the classification result is correct and useful for nature conservation. The primary objective, carried out in 2017 in three areas of Poland, was to determine the minimum percentage cover from which it is possible to identify a target species by RS methods. A secondary objective of this research, related to the requirements of the method, was to optimize the set of training polygons for a target species in terms of the number of polygons and abundance percentage cover of the target species. Our method has to be easy to use, effective, and applicable, therefore the analysis was carried out using the basic set of rasters—the first 30 channels after the Minimum Noise Fraction (MNF) transformation (the mosaic of hyperspectral data from HySpex sensors with spectral range 0.4–2.5 µm) and commonly used Random Forest algorithm. The analysis used airborne hyperspectral data with a spatial resolution of 1 m to perform classification of one invasive and three expansive plants—two grasses and two large perennials. On-ground training and validation data sets were collected simultaneously with airborne data collection. When testing different classification scenarios, only the set of training polygons for a target species was changed. Classification results were evaluated based on three methods: accuracy measures (Kappa and F1), true-positive pixels in subclasses with different species cover and compatibility with field mapping. The classification results indicate that to classify the target plant species at the accepted level, the training dataset should contain polygons with a species cover ranging from 80–100%. Training performed only using polygons with a species characterized by a variable, but lower, cover (20–70%) and missing samples in the 80–100% range, led to a map which was not acceptable because of a high overestimation of target species. We achieved effective identification of species in areas where the species cover is above 50%, considering that ecosystems are heterogeneous. The results of these studies developed a methodology of field data acquisition and the necessity of synchronization in the acquisition of airborne data, and training and validation of on-ground sampling.

## 1. Introduction

At present, a growing threat to natural vegetation by invasive and expansive plant species is observed all over the world. These species intensely enter the natural and semi-natural ecosystems, reducing biodiversity [1,2]. Early monitoring and prompt action at operational levels are therefore necessary to mitigate the negative effect of expansion of invasive and expansive plants on natural ecosystems with high conservation status. An increasing number of studies indicate that remote sensing (RS) and geographic information systems (GIS) are key tools for the identification and effective management of such threats [3,4,5]. A binary map (presence–absence) rather than map of probability is more practical for the end user in nature conservation [6], because of the ability to calculate the area occupied by a species and to enable the monitoring of changes in the area. The possibility of practical usage of the classification depends on the quality of the result map. The accuracy measures evaluate the classification results in a measurable and objective manner. On the other hand, end user expectations do not have to be completely convergent with these accuracy measures. The assessment of the quality of the map performed by the end user as well as expressed by statistical measures takes into account both the underestimation and overestimation effects that may occur simultaneously. Underestimation of the result in a given class is understood as true-positive pixels less than 100% and the overestimation understood as false-positive pixels greater than 0%. In contrast to statistical measures, the weight of the revaluation error is different from the underestimation error in the end user assessment [7]. A great limitation for the practical use of the results in nature conservation is the overestimation error. This error means that the areas where the actual species is not present are indicated on the map as target species. This significantly reduces confidence in the result map. The map is much better received where it presents the place of the main concentration of the species and underestimates the patches with lower density and area.

For RS mapping of a target species to be effective, several factors must be taken into account. It is necessary to adjust the spatial, spectral, and temporal resolution of the data to plant traits of target species to distinguish them from the surrounding natural vegetation [8,9]. In supervised classification, which is often used in species mapping, it is also important to design an effective sampling plan and to collect good-quality reference data [10]. In some cases, this is not difficult because data can be obtained, at least partly, through remote photointerpretation or temporal analysis of digital orthophotos. However, this method is only useful for some species of trees, shrubs, and large perennials forming compact monospecific patches [11]. In other cases, on-ground reference data acquisition is necessary, especially for herbaceous plants characterized by high phenological variability and growing in heterogenous, species-rich ecosystems. This latter kind of reference data acquisition is often labor-intensive, expensive, and sometimes not feasible due to land inaccessibility [12,13]. The percentage cover of herbaceous plants in natural ecosystems changes significantly over intra- and inter-annual time periods. Therefore, it is good practice to synchronize the acquisition of on-ground botanical reference and RS data. Results of analyses conducted on satellite data [14], hyperspectral airborne [15] data, or data provided by unmanned aerial vehicles [16] are very promising in studies of the spread of invasive and expansive species. Their main advantage is the acquisition of data with very high temporal, spectral, and spatial resolution, which is particularly important for the detection of low-height or low-cover-abundance plant species [17,18].

It is typical to use various accuracy measures, such as Overall Accuracy, User and Producer Accuracy or F1 Score and Cohen’s Kappa for the evaluation the classification results. However, high values of these measures do not always indicate a good final map useful for nature conservation; for example, in the case of using too few samples or only those samples containing readily separable classes in the validation dataset, a poor-quality map will be obtained despite satisfactory values of commonly used accuracy measures. Additionally, independent assessment of the results, made by specialists in the field, is much less common. Furthermore, the reliability of some measures is increasingly being undermined [19]. Even more important is to locate reference polygons so that they represent all types of ecosystems occurring in the studied area [20]. 

For RS to be competitive with traditional methods of species monitoring and mapping, it is necessary to minimize the collection of reference data in the field, due to their high cost and often low accessibility of a given area. Algorithms used in mapping have their minimum requirements for the collection scheme and the number of reference polygons [21]. This is especially true for classification, for which it is necessary to collect training polygons representative of each class to be distinguished in a given area. A smaller number of reference polygons, containing only data on the presence of target species, is required for one-class classifiers, otherwise referred to as the detection approach [22]. However, the collection of both presence and absence polygons is recommended for the development of a map acceptable to the end user [23]. Some researchers indicate that the intelligent selection of typical and representative reference polygons, which is the most accurate representation of reality, allows the number of polygons required for correct classification to be reduced [24]. According to other authors [25], the relative proportion of different samples used for training and validation should correspond to the actual incidence of a given class in the studied area. Therefore, in the case of mapping of plant species, not only should the appropriate number and proportion between samples of species and other types of land cover be considered, but also the best representation of their internal variability. For plant species, the source of this variability is, among others, the variation in the percentage cover. In the literature there is little research on the impact of the aforementioned aspects on the results of classification. Therefore, for the practical application of RS methods to species mapping, it is necessary to expand our knowledge in this area.

This study examined the effect of various modifications to the set of species reference polygons on the results of the classification of one invasive and three expansive herbaceous plants. The results of the conducted analyzes will be practically applied to the monitoring of invasive and expansive species in Poland. Therefore, one aspect of experiment design considered the ease of workflow for practical use. For this reason, the well-known Random Forest algorithm, used to classify species and vegetation with a good result and available as free software was applied. Also, the number of spectral features (the first 30 Minimum Noise Fraction bands (MNF) from HySpex the visible and near-infrared (VNIR) and short-wavelength infrared sensors (SWIR) was limited to minimize the number of reference polygons required by the classifier as much as possible [26]. The reference polygon dataset was divided into two classes: target species polygons and background polygons (different ecosystems) according to the requirements of the end user (among others, services of nature conservation management). The objectives of the research were to investigate: (1) What is the impact of species cover in training polygons on classification results? (2) Is it possible to obtain useful for nature conservation classification results with small training dataset? (3) At what percentage cover it is possible to identify a species, using the proposed classification methods? To increase the reliability of the obtained results, the analysis of correctly classified species pixels in three different cover subclasses were used, and compatibility with traditional field mapping was employed along with commonly used in RS accuracy measures (Kappa, F1).

## 2. Materials and Methods 

### 2.1. Study Species

**Figure 1:**
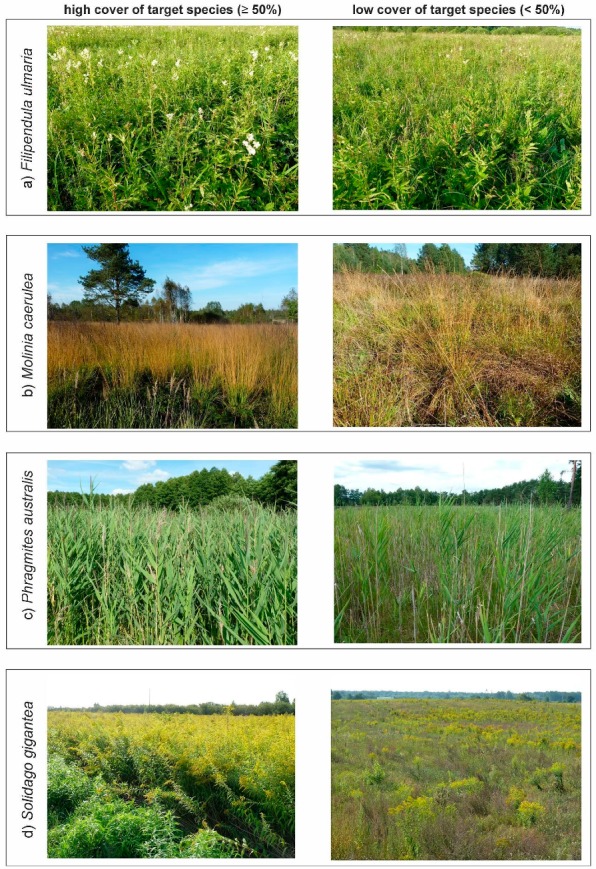
Target species during on-ground sampling, (**a**) *Filipendula ulmaria*, (**b**) *Molinia caerulea*, (**c**) *Phragmites australis*, (**d**) *Solidago gigantea*. (Photo. J. Wylazłowska).

The research was carried out on one alien invasive and three native expansive [27], perennial vascular plants—two grasses (*Molinia caerulea* and *Phragmites australis*) and two large dicotyledons (*Filipendula ulmaria* and *Solidago gigantea*). The research focused on species that are a common threat to valuable non-forest natural habitats in Central Europe. Their expansion involves successively increasing their percentage cover and displacing valuable species; this leads to the reduction of natural high biodiversity. At the early stage of expansion, they may co-occur with many other plants. At later stages of expansion, they finally form compact monospecific patches (with 100% of cover) (Figure 1). They also have morphological and phenological characteristics called functional traits that can be related to spatial and spectral properties different from another species, thus used to discriminate target species from background. At the same time, the similarity of spectral profiles of target species and their background makes classification challenging (Supplementary Materials, Section Spectral Profiles). Each of the target species has been presented against the background of five other plant species. Selected species occur in the main types of non-forest backgrounds, in which the target species may occur. The analysis of the spectral signature profile shows a large similarity between the target species and other species often found in the background class. These characteristics make the selected species valuable objects of classification using RS methods. Here we provided botanical descriptions of all target species.

*Filipendula ulmaria* —FU (meadowsweet) is a herbaceous, perennial, dicotyledonous, tall species typically found in mesic and moist habitats. It naturally occurs in Europe and Asia. The dense, leafy stem reaches a height of 50–200 cm. It blooms from July to August. White flowers are gathered in dense, large inflorescences [28] (Figure 1a). FU grows on wet meadows, in reed beds, and on the shores of water bodies. It is particularly abundant in semi-natural meadow communities, where agriculture use has been discontinued or is occasional. 

*Molinia caerulea*—MC (purple moor-grass) is a perennial, large tussock grass with a wide distribution range covering Europe, Asia, North Africa, and North America. It flowers between July and September, when it grows up to 130–250 cm. The leaves are narrow and delicate, reaching approximately 4–8 mm. In autumn, the whole plant turns a characteristic orange–brown color [29] (Figure 1b). The species occurs mainly in wet meadows, but it can also be found in other habitats, such as forests, heaths, and peat bogs. 

*Phragmites australis*—PA (common reed) is the most widespread flowering plant in the world, occurring throughout North and South America, Europe, Asia, Africa, and Australia [30]. It is a robust, erect, aquatic, or subaquatic perennial grass with culms from 250–450 cm high (in dry localities, much shorter than in aquatic habitats). Its leaves are long (up to 60 cm) and wide (from 0.8 to 6 cm); flowers are gathered in a thick panicle (Figure 1c). The common reed spreads via stolons and rhizomes, and produces dense stands [31,32]. Vegetative propagules are the most important means by which the species propagates and spreads. Colonies expand peripherally by lateral rhizome growth, typically subterranean [30]. The common reed has a very wide ecological spectrum.

*Solidago gigantea*—SG (giant goldenrod) is a tall, erect perennial herb with annual aboveground shoots and persistent belowground rhizomes. Shoots are 5–11 mm in diameter and vary from 30 to 280 cm in height. SG flowers late in the season—between July and November. Inflorescences form pyramidal panicles (Figure 1d). SA is native to North America and is considered to be one of the most aggressive plant invaders in Europe [33,34]. In the secondary range, the goldenrod shows a wide ecological amplitude and habitat spectrum [35,36]. In dry places, the plant co-occurs with other plant species, while in humid places it can form dense colonies developing from its creeping rhizomes and from self-seeding [37]. 

### 2.2. Study Areas 

**Figure 2:**
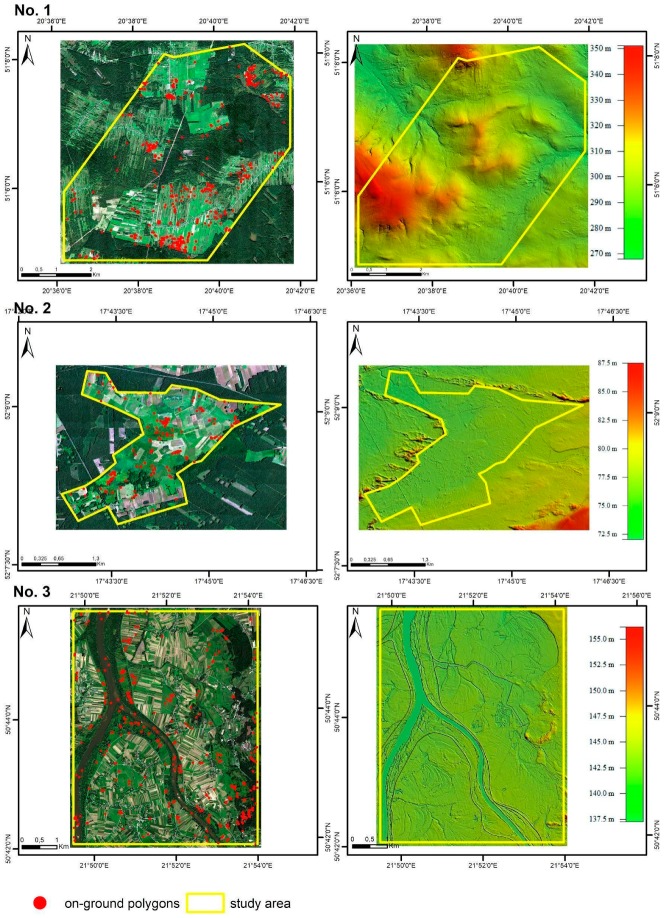
Location of the study areas. Left side: base map: HySpex image in natural color Red, Green, Blue (RGB) composition with on-ground polygon locations. Right side: surface elevation model. Study areas: No.1 “Dolina Krasnej", No.2 “Ostoja Nadwarciańska”, No.3 “Dolina Dolnego Sanu”.

The research was carried out at three areas located in central Poland (Figure 2, Supplementary Materials, Section Study Areas). Study areas are in the warm-summer humid continental climate zone with an average annual temperature of 6–10 °C. The average annual precipitation for the whole of central Poland is approximately 600 mm/yr. Study area No. 1 covers of 40.59 km^2^ and is part of the Special Site of Conservation of Nature 2000 habitats “Dolina Krasnej”, within which one of the main objectives is to preserve *Molinia* meadows on calcareous, peaty, or clayey-silt-laden soils (protected as Natura 2000 habitat). Two species were researched there: FU and MC. Study area No. 2 covers of 10.37 km^2^ and was used to investigate PA. It is a fragment of the Special Site of Conservation of Nature 2000 habitats “Ostoja Nadwarciańska”, which covers part of the Warta River valley. It is one of the best preserved semi-natural landscapes of the lowland river valley in Poland. The area is characterized by the presence of transition mires and quaking bogs, inland salt meadows and *Molinia* meadows on calcareous, peaty, or clayey-silt-laden soils. Area No. 3 is in the confluence of the Vistula and the San River. It covers of 35.45 km^2^ and includes part of the Special Site of Conservation of Nature 2000 habitats “Dolina Dolnego Sanu”. The site was used to research SG. The area is dominated by an agricultural landscape as well as meadow and shrub habitats, including alluvial meadows from the *Cnidion dubii* alliance of high nature conservation value, occurring in river valleys, and lowland hay meadows. The target species pose a threat to native habitats at all the selected areas, including Natura 2000 habitats. Most of these habitats are heterogeneous, species-rich meadows. Only one or two species were selected for each study area, as the most dangerous ones. In most cases, the classification of only one target species in an area is required by end user [7].

### 2.3. Airborne Data Acquisition 

Field and RS data were acquired in 2017. The time of data acquisition was set based on the phenological and structural characteristics of each of the species (Table 1). The peak of development of a target species for a given date, when it is best distinguished from the background, has been determined during HabitARS project [7]. The hyperspectral data were obtained with the HySpex sensor developed by the Norwegian Norsk Elektro Optikk (NEO) company. The HySpex sensor consists of two imaging spectrometers covering spectral ranges of 0.4–0.9 μm (VNIR-1800) and 0.9–2.5 μm (SWIR-384) (Table 2). HySpex VNIR-1800 has 182 spectral bands and HySpex SWIR-384 has 288 spectral bands. There is overlap between the spectral ranges of these two sensors. VNIR-1800 bands from 164 to 182 overlap the spectral range of SWIR-384. Therefore the combined data from two sensors covers the spectral range from 400 to 2500 nanometers in 451 bands (163 VNIR and 288 SWIR). The HySpex instrument was flown on board a Cessna CT206H at an average altitude of 730 m AGL (Above Ground Level), with an airspeed of 59.2 m/s. Orientation of the flights: North-South (NS) or West-East (WE) was adjusted to the azimuth sun angle. When the flight direction is aligned to the solar plane, the Bidirectional Reflectance Distribution Function (BRDF) effect is less affecting the imagery.

Orthorectification and georeferencing was performed in PARGE (ReSe Apps) using standard aircraft in-flight information, such as flight trajectories, altitude and camera metrics in combination with a Digital Surface Model [38]. Next, data from both sensors (VNIR, SWIR) were combined into one hyperspectral data cube, setting the split wavelength to 935 nm and trimming the rows to the field of view of the SWIR imagery. As a result, a data cube with a spatial resolution of 1 m and a pixel position accuracy of RMS = 0.77 m (Root Mean Square) was obtained. The first 430 channels were used in the further processing. Atmospheric correction was performed in ATCOR-4 (ReSe Apps), using the MODTRAN model. The spectral were smoothed using the Savitzky–Golay filter (with a range of 13 channels). Collections of images for each study area were then mosaicked. Each mosaic of hyperspectral data was transformed using the Minimum Noise Fraction transformation (MNF in ENVI version 5.4). The first 30 most informative channels of each MNF mosaic were selected for further analyses. This number was determined based on the MNF eigenvalue plot and visual assessment.

### 2.4. On-Ground Botanical Data for Training and Validation

On-ground botanical reference data were obtained simultaneously with the acquisition of airborne data (year 2017). The botanical fieldwork involved the establishment of reference polygons for the target species and the background. The reference polygon was a circle with a radius of 2 m, which gives about 15 pixels per polygon. Geolocation of reference polygons were recorded using a GNSS (Global Navigation Satellite System) Mobile Mapper 120 with real time differential correction and a measurement accuracy of up to 0.2 m. Simultaneously with obtaining airborne data on-ground training and validations dataset were collected. During the fieldwork, the reference polygons were not split for training the model or for its validation. 

Reference polygons were established for each of the four target species (MC, FU, PA, SG) and for the background class. For each species, 110 reference polygons and 200 background polygons were established, giving a total of 1650 pixels of target species and 3000 pixels of background. All reference polygons were distributed as evenly as possible in the study areas, guided by the actual occurrence of a target species or all given types of ecosystems (as background) in a study area. The reference polygons were collected without spatial bias [23]. The polygons for the target species were in places where the species under study occurred with different percentage cover (from 20 to 100%). The numbers of polygons established for particular cover ranges are presented in Table 3. In addition to the percentage cover, the dominant growth (vegetative/flowering/fruiting) stage for the target species as well as the percentage cover of co-dominant species was recorded for each polygon. The background polygons were set up in such a way to present the entire variability of the surveyed area, including ecosystems in which a target species was recorded or dominated by a species with similar morphology. Considering all polygons in which the target class was not present as background class simplifies the whole process of classification. Due to these assumptions, the reference polygons for the target species and the background carried typical and representative information, which allowed a reduction in the number of polygons required by the Random Forest Classifier [24,25]. 

### 2.5. Field Mapping

Simultaneously with on-ground botanical data acquisition (see Section 2.4), a field map of the distribution of each target species was made for a selected area. For each species, one control area in the shape of a square and a size of 10 ha was chosen. This area was determined in such a way as to represent the full variability of the density of the species in the patches (from 20 to 100%). By using GNSS Mobile Mapper 120, all patches of the target species, with a minimum area of 10 m^2^ and a minimum of 20% land cover, were mapped. In this way, cartographic information was obtained, which became the basis for assessing the correctness of classification.

### 2.6. Random Forest Classification and Accuracy Assessment

Supervised classification with the Random Forest algorithm [39] was applied as a method of classification. The Random Forest classifier has been successfully used in numerous ecological studies, including the mapping of individual plant species [40,41]. The reduction of the number of features from hundreds to tens is recommended in the case of the Random Forest algorithm [26]. Therefore, a dimension reduction step (MNF) was carried out and the first 30 bands selected for classification [26], which also reduces the required number of on-ground training samples. For each result, Kappa and F1 accuracy were calculated [42,43]. The whole classification workflow was scripted in Vegetation Classification Studio [44], using its YAML-based Experiment Definition Language. To facilitate potential comparison of our results with other studies, no specific Random Forest parameter tuning was done, and most parameters were left at the commonly used defaults. There were 100 trees learned for each model, with the Gini criterion used for determining splits, and taking into account the square root of the number of features for each split.

**Figure 3:**
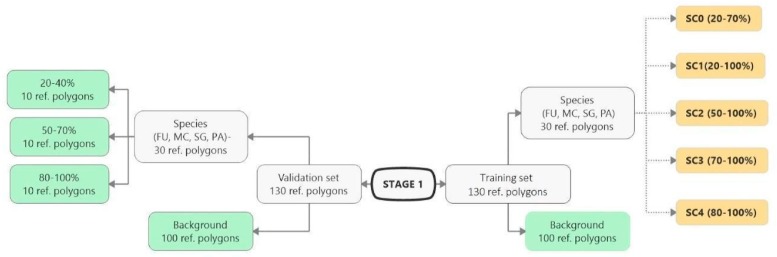
Stage 1 scheme. The number of polygons for the target species and their percentage cover in the polygons and the number of background polygons considered in the training set in each scenario. The group of green rectangles indicates constant and invariable polygons during Stage 1. The group of yellow rectangles indicates scenarios that change during Stage 1, differing in the species percentage cover of in the polygons (FU—*Filipendula ulmaria*, MC—*Molinia caerulea*, PA—*Phragmites australis*, SG—*Solidago gigantea*).

**Figure 4:**
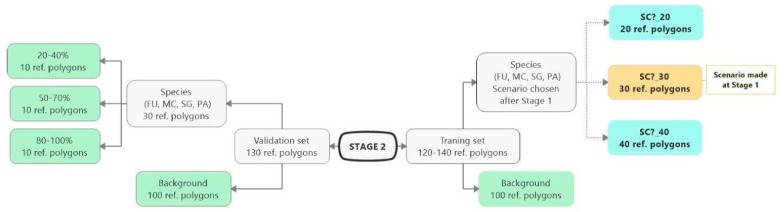
Stage 2 scheme. Changes in the number of species polygons and the number of background polygons taken into account during the training in particular scenarios. The group of polygons with the green background is constant and invariable during Stage 2. The yellow rectangle indicates the best scenario from Stage 1. The group of blue rectangles indicates scenarios, which change during Stage 2, differing in the number of species training polygons (FU—*Filipendula ulmaria*, MC—*Molinia caerulea*, PA—*Phragmites australis*, SG—*Solidago gigantea*).

The classification experiment was divided into three stages (0, 1, 2), which were carried out in sequence. All pixels from given polygon was considered only for training or only for validation to avoid autocorrelation. Stage 0 involved a classification for each target species, in which the set of 110 target species polygons and 200 background polygons were randomly divided into a training and a validation set in a proportion of 50/50. The internal heterogeneity of polygons was not taken into account in this random division. To carry out the next two stages, the reference polygons (both types—target species and background) were permanently divided into a training or validation set. Classification at Stages 1 and 2 differed from each other only in the set of training polygons for the target species (Figure 3 and Figure 4). At Stage 1 the training polygons were selected based on the cover percentage of a target species, while at Stage 2 the number polygons in the training data was considered.

The fixed validation dataset at Stage 1 and 2 for all scenarios consisted of 30 reference polygons for target species and 100 for background polygons. The manually selected validation set was characterized by following criteria: a homogeneous spatial distribution of polygons throughout the study area; 10 target species polygons from each of three range cover classes: 20–40%, 50–70%, 80–100%; the background polygons represented all types of ecosystems occurred in the given study area. 

Since the training and validation set had to meet several criteria, it was not possible to conduct a multiple and random division into training and validation. However, at stages 1 and 2, the relative proportion of target species and background class polygons corresponded to their proportions in the landscape. 110 polygons were collected during on-ground data acquisition, but only 30 was selected to each scenario. 

At Stage 1, the same set of scenarios was created for all four target species, which differed in the range of species percentage cover in the training polygons (scenarios SC0, SC1, SC2, SC3 and SC4), while maintaining a constant number of species and background polygons (Figure 3). The background training set was constant in all scenarios and consisted of 100 polygons which represented all types of ecosystems in the given study area. The set of background polygons was selected manually so that polygons of the same ecosystems were included in the set of training and validation polygons.

Stage 2 followed the completion of Stage 1. Two additional scenarios with 20 and 40 target species training polygons were carried out for the best-rated scenario in Stage 1. The objective of Stage 2 was to check how the change in the number of species training polygons affects the result of classification. The background training polygons and the whole set of validation polygons did not change with respect to Stage 1 and was constant during the classification process at Stage 2. In accordance with this scheme, the classification was performed for all four target species.

Three methods were used to assess whether the classification result was correct and acceptable by end user. The first criterion, used at all stages, were the accuracy measures, i.e., Kappa and F1. At stages 1 and 2, the next criterion was the quantitative analysis of the percentage contribution of correctly classified target species pixels in validation polygons divided into three cover classes: 20–40%, 50–70% and 80–100%. The third criterion was the assessment with compatibility with field mapping (see Section 2.5). All the obtained classification results (Stage 0, Stage 1, Stage 2) were evaluated. The result of the classification for each scenario was compared with the map of the actual occurrence of the species patches mapped during field campaign in the control area. The calculations were made using zonal statistics in ArcGIS (version 10.6), assigning the classification pixels to one of four groups: True Positive (target species pixels classified in actual species patches mapped during field work), False Positive (target species pixels classified in background patches), True Negative (background pixels classified in the background patches) and False Negative (background pixels classified in the target species patches). Each of the classification results was assessed independently, using the three measures described above (Kappa and F1 measure, compatibility with field mapping) and the best classification result was selected on their basis.

## 3. Results 

### 3.1. Classification—Simply Approach (STAGE 0)

**Figure 5:**
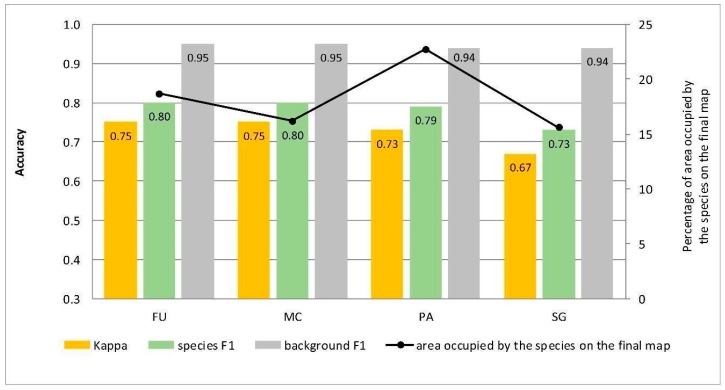
Classification accuracy described by Kappa and F1 measures (columns) and percentage of area occupied by the species on the final map (the black line) for four target species (FU—*Filipendula ulmaria*, MC—*Molinia caerulea*, PA—*Phragmites australis*, SG—*Solidago gigantea*) in the classifications performed at Stage 0.

**Figure 6:**
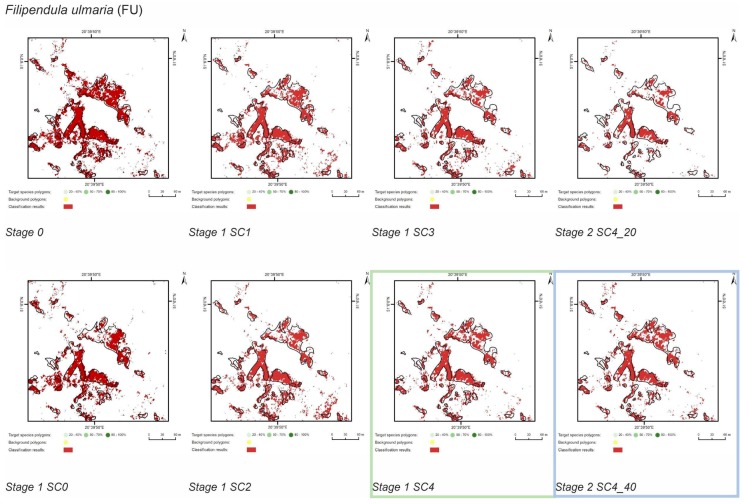
Classification results of *Filipendula ulmaria* (FU) in all stages and scenarios. The black line areas show the target species patches identified during the field mapping. The best scenario for Stage 1 is marked by green line. The best scenario for Stage 2 is marked by blue line.

**Figure 7:**
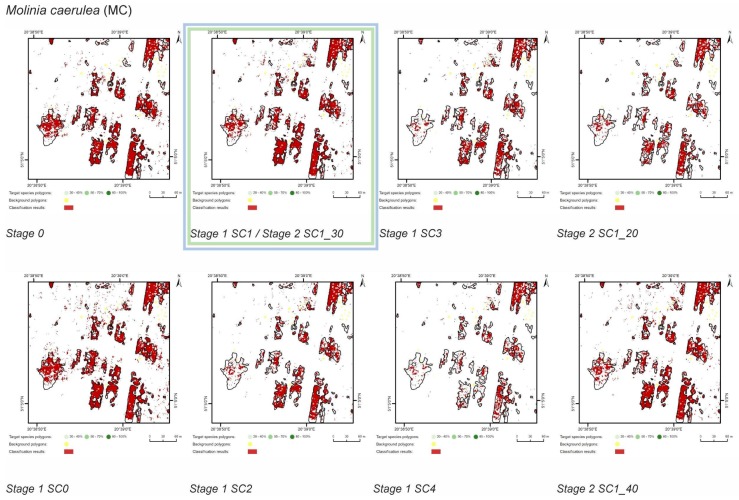
Classification results of *Molinia caerulea* (MC) in all stages and scenarios. The black line areas show the target species patches identified during the field mapping. The best scenario for Stage 1 is marked by green line. The best scenario for Stage 2 is marked by blue line.

**Figure 8:**
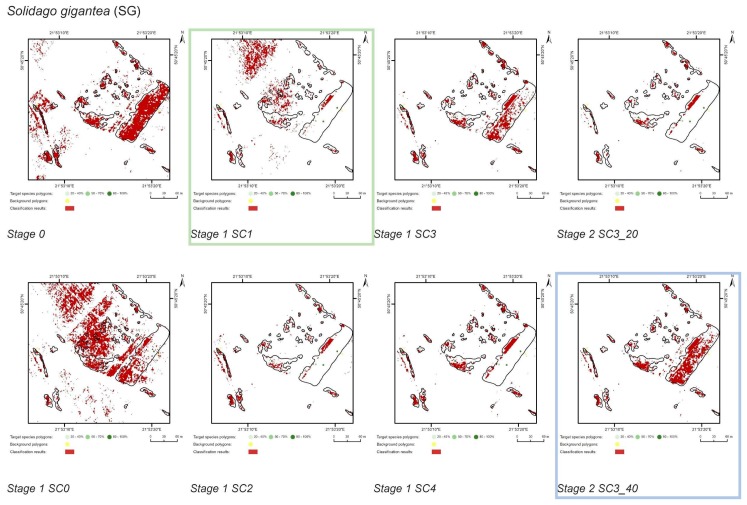
Classification results of *Solidago gigantea* (SG) in all stages and scenarios. The black line areas show the target species patches identified during the field mapping. The best scenario for Stage 1 is marked by green line. The best scenario for Stage 2 is marked by blue line.

**Figure 9:**
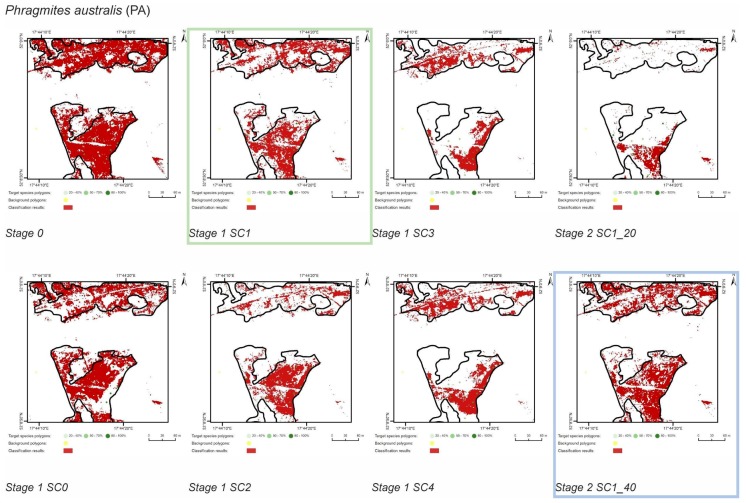
Classification results of *Phragmites australis* (PA) in all stages and scenarios. The black line areas show the target species patches identified during the field mapping. The best scenario for Stage 1 is marked by green line. The best scenario for Stage 2 is marked by blue line.

At Stage 0, relatively high values of Kappa (0.67–0.75) and F1 score (0.73–0.80) were obtained for all target species (Figure 5). At the same time, the compatibility with field mapping and the classification results on the control area showed an overestimation in the target species classes, at the level between 10.7% for PA to 54.7% for FU false-positive pixels (Table 4). The results for all target species in this scenario were not useful for nature conservation. Although they were characterized by a high degree of target species detection, they also had too much overestimation in the background patches (Figure 6, Figure 7, Figure 8 and Figure 9).

### 3.2. Classification—Various Cover of Target Species in Training Polygons (STAGE 1)

**Figure 10:**
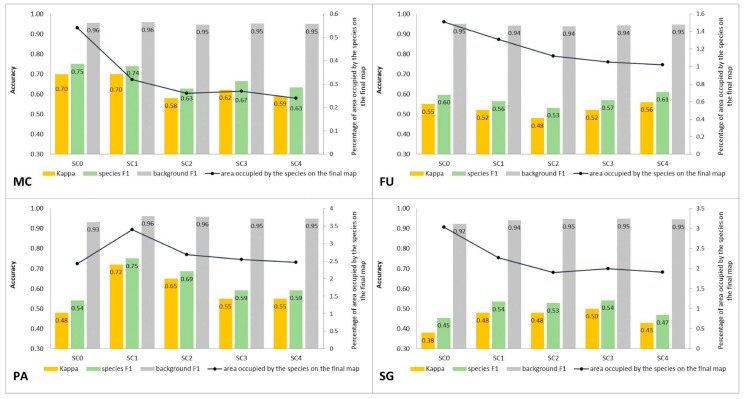
Classification accuracy described by Kappa and F1 measures (columns), percentage of area occupied by the target species on the final map (the black line) for four target species (FU—*Filipendula ulmaria*, MC—*Molinia caerulea*, PA—*Phragmites australis*, SG—*Solidago gigantea*) in five classification scenarios performed at Stage 1.

**Figure 11:**
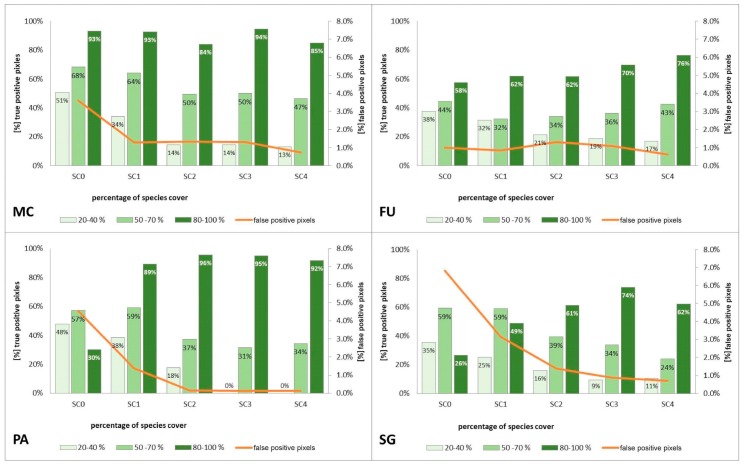
Percentage of correctly classified pixels (columns) and percentage of false-positive pixels (the orange line) in the set of validation polygons for four target species (FU—*Filipendula ulmaria*, MC—*Molinia caerulea*, PA—*Phragmites australis*, SG—*Solidago gigantea*) in four scenarios of Stage 1.

The purpose of SC0 (20–70%) was to check the possibility of correctly mapping target species in the absence of training polygons with high cover of target species (80–100%). The results obtained for all target species were useless for end user, although high Kappa (>0.5) and F1 (>0.6) values were obtained for two species (MC, FU; Figure 10). For three species: FU, MC, and SG, the results suggested a significant overestimation (high number of false-positive pixels—from 24.3% to 90.9%), (Figure 6, Figure 7 and Figure 8 and Table 4). However, in the case of the PA class, the result was underestimated (Figure 10). In most cases, polygons with low species cover were correctly identified and the background was incorrectly identified as the target species. The results indicate that SC0 has the best capacity for species mapping at an early stage of their expansion (20–40% species cover in a polygon), but at the same time it significantly overestimates a target species (Figure 11 and Table 4). 

The SC1 (20–100%) scenario was the best as assessed by all three methods (Table 5) for the two grass species characterized by a similar growth strategy, MC and PA. Also, the values of percentage of area occupied by the target species on the final map and expected percentage of study area occupied by the target species were quite similar to the estimated area (Figure 10). The advantage of SC1, compared to other scenarios for grass species, is more effective in correctly classifying the target species with lower cover, i.e., 20–40% and 50–70% (Figure 11), while also possessing a high accuracy in the cover class of 80–100%. Only grasses were not overestimated (MC 13% and PA 5% false-positive pixels on the control area) (Table 4), which is reflected in the results for SG (36%) and FU (46%) (Figure 6 and Figure 7). For large perennials (FU and SG), the SC1 scenario was not considered useful for nature conservation for two reasons; firstly, their overall abundance was overestimated; secondly the poor classification performance: for FU in the range of 20–40% and 50–70%, while for SG in the range of 20–40% and 80–100% (Figure 11). The SC2 (50–100%) scenario yielded only average validation scores. SC3 (70–100%) was the best as assessed by all three methods (Kappa and F1 measure, compatibility with field mapping) for one of the target perennial species, SG. This scenario mainly correctly classifies the target species, SG, with higher cover—more than 80% (Figure 10). In other scenarios, SG was correctly categorized with lower accuracy, especially in the 80–100% cover range, and the result of classification reached lower values of Kappa and F1. For SG, the classification in SC3 was better than SC4 by comparing results in the control area due to a higher percentage of true-positive pixels (36% to 22%) (Table 4). SC4 (80–100%) gave the best results for the other perennial species, FU. Only this scenario allowed the correct classification of target species polygons in the range of 50–70% and 80–100%, as well as produces the highest values of Kappa and F1 score. The results for SG, MC, and PA were not useful for end user in SC4 (Figure 7, Figure 8 and Figure 9). This was due to a worse ability to correctly classify species with lower cover classes, i.e., 20–40% and 50–70% (Figure 11). For these three target species, the results in the control area were significantly underestimated (they reached low value of true-positive pixels from 22 to 36% (Table 4).

### 3.3. Classification—Various Number of Target Species Training Polygons (STAGE 2)

The purpose of Stage 2 was to check how the change in the number of species training polygons affects the result of classification. First, the best classification obtained at Stage 1 was selected. Then, the set of training polygons for each species were modified to increase and decrease the number of training polygons by 10. As a result, three classification scenarios were created with the following numbers of training polygons for a target species: 20, 30, and 40.

**Figure 12:**
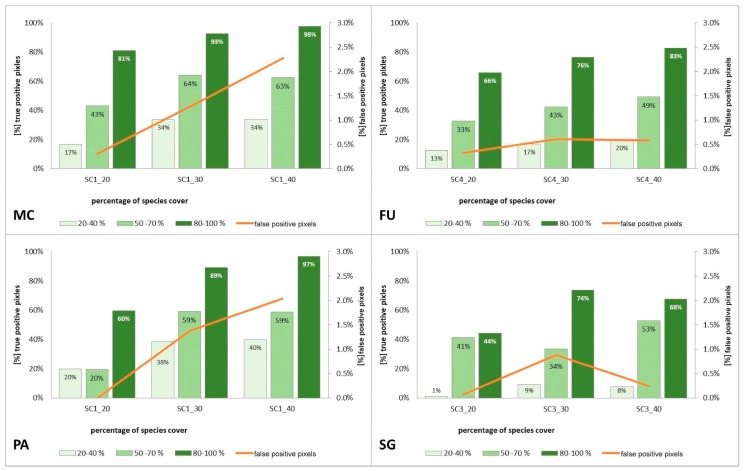
Percentage of correctly classified pixels (columns) and percentage of false-positive pixels (the orange line) in the set of validation polygons for four target species (FU—*Filipendula ulmaria*, MC—*Molinia caerulea*, PA—*Phragmites australis*, SG—*Solidago gigantea*) in three scenarios of Stage 2.

**Figure 13:**
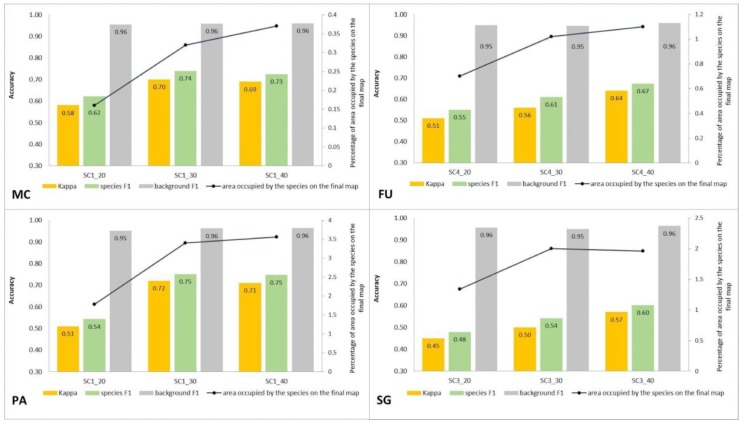
Classification accuracy described by Kappa and F1 measures (columns), percentage of area occupied by the target species on the final map (the black line) for four target species (FU—*Filipendula ulmaria*, MC—*Molinia caerulea*, PA—*Phragmites australis*, SG—*Solidago gigantea*) in three classification scenarios performed at Stage 2.

Compared to the initial scenarios of Stage 1 (30 species training polygons), none of the scenarios with 20 species polygons in the training set yielded a good classification result of classification when considering all three methods of quality assessment. For all target species, the scenario with a reduced number of species training polygons achieved the lowest Kappa and F1 values compared to other scenarios of Stage 2, at the same time compatibility with field mapping (Figure 6, Figure 7, Figure 8 and Figure 9 and Table 4), analysis of correctly classified pixels (Figure 12) indicated an underestimation of classified species, especially in the cover range of 20–40% and 50–70%. At the same time, all three criteria showed very small differences between the scenarios based on 30 and 40 species training polygons, while the correctly classified results indicated the possibility of classifying a species in patches with PA and MC cover above 50%, and FU and SG cover above 70% (Figure 13). The best results for MC were obtained with the scenario based on 30 species training polygons, taking into an account two of the three measures of the assessment: accuracy measures and compatibility with field mapping (Table 6). MC is correctly classified only in cases with at least 50% cover in a polygon; below this value the result is not acceptable, only 34% of correctly classified pixels in the 20–40% class (Figure 12). On the other hand, the best scenario for FU, PA, and SG was the one based on 40 species training polygons, reaching the highest values for all evaluated parameters (Table 6). Differences in the classification between SC_30 and SC_40 were not significant for FU, PA, and SG. The analysis of the control area showed that SC_40 was characterized by slightly better classification of the target species (true-positive pixels) and at the same time stable and less overestimation (false-positive pixels) (Table 4).

## 4. Discussion

### 4.1. Effect of the Species Percentage Cover in the Training Dataset on the Classification Results

The effect of the percentage cover of herbaceous species within training polygons on the results of identification using RS data has been very seldom analyzed in the literature. In some cases, this factor was not taken into account because researchers either used reference training data not obtained at the similar time as airborne data [45] or obtained information through analysis of digital orthophotos [46]. Publications often provide information on changes in the percentage cover of a species, for which the reference was collected [47,48], but seldom include information on the distribution of the number of study plots differing in the species cover [49]. Furthermore, a significant discrepancy between the date of airborne and botanical data acquisition reduces the reliability of the conclusion [50]. Significant changes, related to the dynamics of growth and phenology, occur in the cover of individual herbaceous plant species during the growing season as well as in the successive years [51]. In the present research, an approach has been applied which takes into account both the time convergence of data acquisition (Table 1) as well as detailed information on the species cover in the reference polygons (Table 3). This approach has enabled a comprehensive analysis of the effect of species cover in training polygons on the classification results and has indicated a significant influence on the results of Random Forest classification. The lack of pre-selection of training polygons at Stage 0 (Figure 5), although numerous, resulted in poor-quality classification results, while maintaining high values of Kappa and F1 (Figure 5). It should be emphasized that values obtained for these typical accuracy measures at Stage 0 were much higher than those obtained at further stages of the experiment, which calls into question the assessment of the reliability of classification outcomes by means of these measures alone. This is confirmed by previous studies questioning the reliability of, among other things, Kappa measures [19], which are commonly used in RS of the environment. At each of the analyzed sites, the area covered by false-positive species pixels at Stage 0 was significantly larger than that obtained by the best-rated scenario (Figure 5). The results clearly indicate that when designing a sampling plan and selecting training polygons, the variability in cover and spatial patterns of a species, as well as the composition of co-occurring vegetation in a polygon should be considered. This is supported by the reports of other researchers who advocate the need to sample the ecosystem as faithfully and effectively as possible [52] or risk a significant overestimation in species abundance.

The impact of different scenarios on the classification outcomes varied depending on the species, but general trends can be observed. For all target species, training with polygons characterized by a lower species cover in a polygon (20–70%), not allowing for spectral purity of target species pixels, resulted in more effective identification at an early stage of expansion, i.e., in areas where a target species achieved lower cover-abundance. At the same time, however, the result obtained during traditional field mapping was rated worst due to the overestimation of a species (high number of false-positive pixels). This result, due to the strong overestimation, is characterized by a low applicability in the management of nature conservation. This overestimation is the result of no unique background species compositions which co-exist with target species. The same background species can occur in both target and background polygons and can achieve high cover when the target species has low cover. On the other hand, training consisting of polygons with only high species cover (80–100%) resulted in the underestimation of a species with a low cover, but the result was assessed as more favorable by the end user as it was not affected by an unfavorable overestimation. Therefore, when adopting a strategy for reference data acquisition, one should be aware which result is better for the end user and then design the acquisition of reference data.

The results also indicate that to obtain the best results, it is necessary to adjust the selection of training polygons to traits of a given species. Effective classification requires adaptation of spatial, spectral and temporal resolution of data to match traits of the target plant species [8,9]. In the case of the analyzed species, the classification results were affected not only by phenological but also structural traits and their variability in the surveyed areas, as well as the spectral characteristics of a given species. The analyzed dicotyledonous perennials (SG, FU) are characterized by the small size of individuals, especially at an early stage of expansion. Furthermore, their shoots usually intertwine with shoots of other plant species, which means that even with a relatively high degree of cover in a polygon and the optimal data acquisition date, their signature strongly affected by the reflectance of other plants growing in the same polygon and will therefore possess a smaller spectral separation from the background class [53]. For this reason, to obtain reliable results of classification for species with similar spectral characteristics (Supplementary Materials, Section Spectral Profiles), mainly polygons with a high degree of cover (up to 70–100%) should be used for training. Different results were obtained for MC, which is a grass forming compact, relatively large tussocks and large amounts of necromass [24]. In the case of MC, successful results of classification were obtained using training polygons with a cover of 20–100% (Figure 10).

### 4.2. Percentage Cover of Target Species that Enables Its Identification

In the studies of species classification by RS, it is very important to analyze the minimum target species cover that can be successfully detected, as it determines the limit from which proper mapping is possible. This knowledge allows us to deduce the extent to which it is possible to apply this method to map the early stages of an invasion. At the same time, there are only a few examples of studies that assess the ability to map smaller cover fractions [53,54]. One means of understanding the possibility of successful classification depending on the species cover via the creation of separate classes of validation polygons [45]. However, the disadvantage of this approach is that the observed inaccuracies often come from confusion between different density classes, so it is not possible to draw robust conclusions. In the present study, one class was used in the validation set and the quantitative analysis of the contribution of correctly classified species pixels within the validation polygons was carried out, divided into three cover classes: 20–40%, 50–70% and 80–100%. In addition, not only the standard measures of accuracy were used to assess the quality of the results, but also the percentage of area occupied by the species on the final map and comparison with the results of traditional field mapping. The analysis of correctly classified pixels in the validation polygons by percentage cover indicates that it is possible to quantify identify target species in heterogenous ecosystems when the percentage contribution of the species is at least 50% (Figure 11). A training set consisting of polygons with species cover in the range 20–70% allowed for slightly more effective mapping at an early stage (low cover) of expansion of a target species. At the same time, it caused a significant overestimation of a species in the study area, which precludes the practical application of this result. In addition, it should be noted that the high value of typical accuracy measures often did not correspond with the results of traditional field mapping. This was particularly evident during the analysis of the results of Stage 0 and Stage 1 in SC0 for the species FU, MC, and SG (Figure 5 and Figure 10 and Table 4). These scenarios yielded high values of Kappa and F1, which would lead to an assumption of a correct and useful classification, obtained the lowest compatibility with field mapping (Figure 10 and Table 4). Other studies also indicate that there are discrepancies between the obtained accuracy measures and the actual assessment of the map of the identified species [6].

There are several examples in the literature indicating the possibility of identifying plant species even at very low degrees of cover [17]. However, for the results to be reliable and reproducible, a limit (threshold) of 30–40% cover should be adopted [53]. Our research indicates a limit of 50% species cover, within the context of the specific methodology of this study. We hypothesize that there may be a characteristic vegetation signature associated with low target species cover (below 50%) that is significantly different from all other background-only signatures. If this hypothesis were correct, a high ratio of true positives to false positives could be achieved for the species classes with low percentage cover. If this hypothesis is not correct, the classification would be based only on the target species signature alone, not the whole vegetation signature, then the classification would be successful only for the species with higher than, in this case, 50% cover. In addition, the classification results depend on the traits of a target species and its ecosystem. Therefore, trees, shrubs, and large perennials, as well as plants occurring as weeds in cultivated fields, are easier to identify [55,56]. Significantly worse results are obtained in the case of small herbaceous plants occurring in heterogeneous ecosystems [57]. 

In addition, other researchers argue that the interpretation of accuracy measures is usually the only way to evaluate results. The reliability of their values is intrinsically associated with the quality and the number of polygons used in validation, which is often insufficient and does not always cover all major types of land cover [58]. The results of this study indicate the necessity of close cooperation between end user and RS specialists at each stage of the research. Knowledge of a given area, and appropriate verification of the results on maps, are crucial for a meaningful assessment of the classification results. Particular attention should be paid to the appropriate selection of background polygons, not limited to the basic on-ground classes, but also taking into account, among other things, the ecosystems in which a target species may occur. 

### 4.3. Impact of the Number of Target Species Training Polygons on the Result of Classification

According to current literature, the relative proportion of samples representing different classes used for training and validation should correspond to the actual incidence of a given class in the studied area [26]. Violation of these proportions, for example, over-representation of the target class in the training data can lead to an overestimation of a class. Examples are the results of *Fallopia japonica* identification using Random Forest [46] and *Tamarix* sp. identification using the Maximum Entropy model [50]. The results of our research confirm that too many training polygons of a species occupying only a small part of in the surveyed area (<5%) leads to an overestimation of the abundance of the species in the classification result when using the Random Forest algorithm (Figure 5). The problem of underestimation in all cover classes of target species occurred when the number of training polygons of a target species was too small. A useful classification was not achieved for any target species when using 20 training polygons (Figure 12), regardless of whether the study area covered 10 km^2^ (No. 2) or 40 km^2^ (No. 1). The best results of classification were obtained for the set of 30 target training polygons in the case of MC and 40 target training polygons for FU, SG, PA (Table 6). Depending on the area, the training polygons occupied from 0.01% to 0.07% of the surveyed area (calculated area covered by the target species polygons). It can therefore be assumed that a set of polygons, optimized in terms of the percentage cover of a species, allows for a correctly assessed classification with a significantly lower sampling rate compared to 1–2% of the area described in the literature [59]. The correct result of identification on a comparable set of training polygons was achieved with the use of the Mixture Tuned Matched Filtering (MTMF) algorithm [58] when mapping *Cardaria draba*. It can be concluded that the results confirm the high sensitivity of the Random Forest algorithm to the problem of class balance [46]. Therefore, it seems justified to further test the Random Forest algorithm, accounting for imbalanced classes to identify natural phenomena of unknown intensity during sampling design [60]. The use of such an algorithm may lead to better classification results when the actual abundance of a target species in in the surveyed area is not known at the outset. 

### 4.4. Applicability of the Obtained Results

One of the limitations of applying RS are high costs of field data acquisition [10,61]. This research indicates that if polygons are effectively selected, the classification of an invasive or expansive species can be carried out over 40 km^2^ with the use of 30–40 species polygons for training, provided a species occupies less than 5% of the surveyed area [7]. This may lead to a reduction in the duration of on-ground botanical work, with an associated reduction in costs, and at the same time an increase in the temporal consistency of airborne and field data. It is recommended to synchronize on-ground botanical data acquisition with airborne data acquisition, since this minimizes the risk of changes in reference polygons, such as changes in the percentage cover of a species through its growth, extinction, mowing or overgrowing [57]. The results also indicate that the data classification algorithm selected has its restrictions related to the species cover limit below which a species is not reliably detectable in a polygon (Figure 11 and Figure 12). The method adopted enables the reliable identification of a species with a cover above 50% in heterogeneous ecosystems. In the case of lower cover, the obtained accuracies exclude the possibility of using the result for species monitoring. This is particularly important when monitoring alien invasive or expansive species where early detection (with low cover) of a species is more likely to result in effective control [3]. 

## 5. Conclusions

1. To classify the species correctly, the training dataset should contain polygons with a species cover ranging from 80–100%. Training performed only on polygons with a species characterized by a varied but lower cover-abundance (20–70%), but missing samples in the range 80–100%, leads to a high overestimation of species in the background class. Such a result of classification prohibits its practical use for species mapping. 

2. The ability to identify a species depends on its morphological and phenological traits. The results indicate that in the case of large grasses (i.e., *Phragmites australis*, *Molinia caerulea*), the best classification results are obtained when training polygons include species cover in range 20–100%. In the case of large dicotyledonous perennials (i.e., *Solidago gigantea*, *Filipendula ulmaria*), the optimal cover content in training polygons should include a narrower range with high cover, greater than 70% (Figure 10 and Table 5).

3. The use of a training scenario adapted to the traits of a target species allows effective identification of that species in areas where the actual species cover is above 50%, for heterogeneous, species-rich ecosystems. The mapping results above these cover fractions are sufficiently reliable to be further used in nature conservation.

4. It is possible to identify a target species that occupies up to 5% of a study area (10 to 40 km^2^) using a training dataset covering less than 0.07% of the surveyed area.

5. Multi-criteria evaluation of classification results indicates an inconsistency between different methods of assessing the correctness of classification results. The discrepancy was found when comparing Kappa and F1 values with results of traditional field mapping. Bearing in mind the final application of the research, compatibility with field mapping must be an integral part of species mapping by RS methods. This problem results from the lack of the possibility of sufficiently dense background sampling for validations in large area with very heterogeneous ecosystems.

## Figures and Tables

**Table 1:** Characteristics of the field and remote sensing data for each study area. Orientation of flight: North-South (NS), West-East (WE).

Area	No. 1	No. 1	No. 2	No. 3
Target species	*Molinia caerulea* (MC)	*Filipendula ulmaria* (FU)	*Phragmites australis* (PA)	*Solidago gigantea* (SG)
Flight dates and start of field sampling	27 September 2017	07 July 2017	16 July 2017	09 September 2017
End of field sampling	29 September 2017	13 July 2017	18 July 2017	19 September 2017
Dominant development phase of target species	fruiting	flowering	flowering	flowering
Phenological and structural traits of plants during acquisition	Plants form a dense tussock, floral stems become pinkish–orange as the leaves turn yellow	Plants form a bushy clump, bearing sprays of creamy–white flowers	Plants form dense stands that include flowering bushy panicles and standing dead stems from previous year’s growth	Plants form a bushy mound of deep-green leaves, bearing large clusters of golden-yellow flowers
Number of established target species reference polygons	110	110	110	110
Number of established background polygons	200	200	200	200
Data collection area [km^2^]	40.59	40.59	10.37	35.45
Number of flight lines	26	25	11	20
Orientation of flight	NS	WE	WE	NS

**Table 2:** Flight parameters and hyperspectral data.

Sensor Type	Data Parameters	Flight Lines Overlap	Swath Width
HySpex VNIR-1800 0.4–0.9 µm	GSD 0.49 [m]	35 [%]	440 m
HySpex SWIR-384 0.9–2.5 µm	GSD 1.07 [m]	30 [%]	410 m

GSD—Ground Sample Distance, the difference in the flight line overlap between two sensors is due to the different Field of View (FOV)—VNIR-1800 has FOV equal 34 degrees and SWIR-384 FOV is 32 degrees. Hence the effective overlap is the one of the SWIR-384 because the VNIR-1800 images are trimmed to the common coverage.

**Table 3:** The number of polygons established for each target species.

Number of the Target Species Reference Polygons and Background Reference Polygons		
Percentage Cover of the Target Species		
20–40%	50–70%	80–100%
30	30	50

**Table 4:** Division of the classification results from each stage and scenario to the area of the target species and their background and to the percentage of True Positive, False Positive, True Negative, and False Negative in the control area selected for each target species (FU—*Filipendula ulmaria*, MC—*Molinia caerulea*, PA—*Phragmites australis*, SG—*Solidago gigantea*).

Target Species	Stage and Scenario	Control Area of Target Species [m^2^]	Control Area of Back-Ground [m^2^]	True Positive [m^2^]	False Positive [m^2^]	True Negative [m^2^]	False Negative [m^2^]	True Positive [%]	False Positive [%]	True Negative [%]	False Negative [%]
MC	Stage 0	14189	85667	9784	1946	83739	4387	69	14	98	31
	Stage 1 SC0			10840	3449	82236	3331	76	24	96	23
	Stage 1 SC1			9812	1774	83911	4359	69	13	98	31
	Stage 1 SC2			7578	878	84807	6593	53	6	99	46
	Stage 1 SC3			6953	694	84991	7218	49	5	99	51
	Stage 1 SC4			5102	460	85225	9069	36	3	99	64
	Stage 2 SC1_20			6123	402	85283	8048	43	3	100	57
	Stage 2 SC1_30			9812	1774	83911	4359	69	13	98	31
	Stage 2 SC1_40			9343	1737	83948	4828	66	12	98	34
FU	Stage 0	10797	89203	8134	5910	83149	2663	75	55	93	25
	Stage 1 SC0			6486	4406	84653	4311	60	41	95	40
	Stage 1 SC1			7102	5006	84053	3695	66	46	94	34
	Stage 1 SC2			6679	4590	84469	4118	62	43	95	38
	Stage 1 SC3			6287	3454	85605	4510	58	32	96	42
	Stage 1 SC4			6457	3126	85933	4340	60	29	96	40
	Stage 2 SC4_20			4684	1482	87577	6113	43	14	98	57
	Stage 2 SC4_30			6457	3126	85933	4340	60	29	96	40
	Stage 2 SC4_40			6453	3057	86002	4344	60	28	96	40
SG	Stage 0	11477	88523	7076	3398	84981	4401	62	30	96	38
	Stage 1 SC0			4208	10431	77948	7269	37	91	88	63
	Stage 1 SC1			1488	4128	84251	9989	13	36	95	87
	Stage 1 SC2			1916	581	87798	9561	17	5	99	83
	Stage 1 SC3			4061	665	87714	7416	35	6	99	65
	Stage 1 SC4			2575	568	87811	8902	22	5	99	78
	Stage 2 SC3_20			1636	211	88168	9841	14	2	100	86
	Stage 2 SC3_30			4061	665	87714	7416	35	6	99	65
	Stage 2 SC3_40			4770	638	87741	6707	42	6	99	58
PA	Stage 0	33106	66894	24417	3534	63216	8689	74	11	95	26
	Stage 1 SC0			18279	3409	63341	14827	55	10	95	45
	Stage 1 SC1			15426	1700	65050	17680	47	5	97	53
	Stage 1 SC2			10979	657	66093	22127	33	2	99	67
	Stage 1 SC3			8604	356	66394	24502	26	1	99	74
	Stage 1 SC4			10450	473	66277	22656	32	1	99	68
	Stage 2 SC1_20			3904	315	66435	29202	12	1	99	88
	Stage 2 SC1_30			15426	1700	65050	17680	47	5	97	53
	Stage 2 SC1_40			16393	1532	65218	16713	50	5	97	50

Green color—the best scenario for Stage 1, blue color—the best scenario for Stage 2.

**Table 5:** A comparison of the three measures with the best independently chosen scenario of for each measure for Stage 1.

Target Species	RS Accuracy Measures (Kappa, F1)	Correctly Classified Species Pixels (%)	Compatibility with Field Mapping (Control Area)	Chosen Scenario
*Molinia caerulea* (MC)	SC1	SC1	SC1	SC1
*Filipendula ulmaria* (FU)	SC4	SC4	SC4	SC4
*Solidago gigantea* (SG)	SC3	SC3	SC3	SC3
*Phragmites australis* (PA)	SC1	SC1	SC1	SC1

**Table 6:** A comparison of the three measures with the best independently chosen scenario of for each measure for Stage 2.

Target Species	RS Accuracy Measures (Kappa, F1)	Correctly Classified Species Pixels (%)	Compatibility with Field Mapping	Chosen Scenario
*Molinia caerulea* (MC)	SC1_30	SC1_40	SC1_30	SC1_30
*Filipendula ulmaria* (FU)	SC4_40	SC4_40	SC4_40	SC4_40
*Solidago gigantea* (SG)	SC3_40	SC3_40	SC3_40	SC3_40
*Phragmites australis* (PA)	SC1_30	SC1_40	SC1_40	SC1_40

## Supplementary Materials

The following are available online at https://www.mdpi.com/1424-8220/19/13/2871/s1.

## References

[1] Hejda M., Pyšek P., Jarošík V. (2009). Impact of invasive plants on the species richness, diversity and composition of invaded communities. J. Ecol..

[2] Carey M.P., Sanderson B.L., Barnas K.A., Olden J.D. (2012). Native invaders—Challenges for science, management, policy, and society. Front. Ecol. Environ..

[3] Bradley B.A. (2014). Remote detection of invasive plants: A review of spectral, textural and phenological approaches. Biol. Invasions.

[4] Huang C.-Y., Asner G.P. (2009). Applications of remote sensing to alien invasive plant studies. Sensors.

[5] Joshi C.M., de Leeuw J., van Duren I.C. Remote sensing and GIS applications for mapping and spatial modelling of invasive species. Proceedings of the ISPRS Congress: Geo-Imagery Bridging Continents 2004.

[6] Skowronek S., Asner G.P., Feilhauer H. (2017). Performance of one-class classifiers for invasive species mapping using airborne imaging spectroscopy. Ecol. Inform..

[7] MGGP Aero (2016). Auxiliary Work in WP6 under the Programme “Natural Environment, Agriculture and Forestry” BIOSTRATEG II.: The Innovative Approach Supporting Monitoring of Non-Forest Natura 2000 Habitats, Using Remote Sensing Methods (HabitARS).

[8] Müllerová J., Brůna J., Bartaloš T., Dvořák P., Vítková M., Pyšek P. (2017). Timing is important: Unmanned aircraft vs. Satellite imagery in plant invasion monitoring. Front. Plant Sci..

[9] Niphadkar M., Nagendra H. (2016). Remote sensing of invasive plants: Incorporating functional traits into the picture. Int. J. Remote Sens..

[10] Baldeck C.A., Asner G.P. (2014). Improving remote species identification through efficient training data collection. Remote Sens..

[11] Aneece I., Epstein H. (2017). Identifying invasive plant species using field spectroscopy in the VNIR region in successional systems of north-central Virginia. Int. J. Remote Sens..

[12] Dubula B., Tesfamichael S.G., Rampedi I.T. (2016). Assessing the potential of remote sensing to discriminate invasive Asparagus laricinus from adjacent land cover types. South Afr. J. Geomat..

[13] Pelletier C., Valero S., Inglada J., Champion N., Sicre C.M., Dedieu G. (2017). Effect of training class label noise on classification performances for land cover mapping with satellite image time series. Remote Sens..

[14] Royimani L., Mutanga O., Odindi J., Dube T., Matongera T.N. (2018). Advancements in satellite remote sensing for mapping and monitoring of alien invasive plant species (AIPs). Phys. Chem. Earth Parts A/B/C.

[15] Peerbhay K., Mutanga O., Ismail R. (2016). The identification and remote detection of alien invasive plants in commercial forests: An Overview. S. Afr. J. Geomat..

[16] Kaneko K., Nohara S. (2014). Review of effective vegetation mapping using the UAV (Unmanned Aerial Vehicle) method. J. Geogr. Inf. Syst..

[17] Glenn N.F., Mundt J.T., Weber K.T., Prather T.S., Lass L.W., Pettingill J. (2005). Hyperspectral data processing for repeat detection of small infestations of leafy spurge. Remote Sens. Environ..

[18] Fassnacht F.E., Latifi H., Stereńczak K., Modzelewska A., Lefsky M., Waser L.T., Straub C., Ghosh A. (2016). Review of studies on tree species classification from remotely sensed data. Remote Sens. Environ..

[19] Pontius R.G., Millones M. (2011). Death to Kappa: Birth of quantity disagreement and allocation disagreement for accuracy assessment. Int. J. Remote Sens..

[20] Castaldi F., Chabrillat S., van Wesemael B. (2019). Sampling strategies for soil property mapping using multispectral sentinel-2 and hyperspectral EnMAP satellite data. Remote Sens..

[21] Stehman S.V. (2009). Sampling designs for accuracy assessment of land cover. Int. J. Remote Sens..

[22] Manolakis D., Shaw G. (2002). Detection algorithms for hyperspectral Imaging applications. IEEE Signal Process. Mag..

[23] Phillips S.J., Dudi’k M., Dudi’k D., Elith J., Graham C.H., Lehmann A., Leathwick J., Ferrier S. (2009). Sample selection bias and presence-only distribution models: Implications for background and pseudo-absence data. Ecol. Appl..

[24] Foody G.M., Mathur A. (2004). Toward intelligent training of supervised image classifications: Directing training data acquisition for SVM classification. Remote Sens. Environ..

[25] Millard K., Richardson M. (2015). On the importance of training data sample selection in Random Forest image classification: A case study in peatland ecosystem mapping. Remote Sens..

[26] Mather P.M. (1999). Computer Processing of Remotely-Sensed Images: An Introduction.

[27] Pyšek P., Richardson D.M., Rejmánek M., Webster G.L., Williamson M., Kirschner J. (2004). Alien plants in checklists and floras: Towards better communication between taxonomists and ecologists. Taxon.

[28] Ball P.W., Tutin T.G., Heywood V.H., Burges N.A., Moore D.M., Valentine D.H., Walters S.M., Webb D.A., Chater A.O., DeFilipps R.A. (1972). Flora Europaea.

[29] Taylor K., Rowland A.P., Jones H.E. (2001). Molinia caerulea (L.) Moench. J. Ecol..

[30] Shaltout K.H., Al-sodany Y., Eid E.M. (2006). Biology of Common Reed Phragmites Review and Inquiry.

[31] Täckholm V., Täckholm G., Drar M. (1941). Flora of Egypt.

[32] Holm L.G., Plucknett D.L., Pancho J.V., Herberger J.P. (1977). Phragmites australis (Cav.) Trin. (= P. communis Trin.) and Phragmites karka (Retz.) Trin. The World’s Worst Weeds “Distribution and Biology”.

[33] Weber E., Jacobs G. (2005). Biological flora of central Europe: Solidago gigantea Aiton. Flora.

[34] Capek M. (1971). The possibility of biological control of imported weeds of the genus Solidago L. in Europe. Acta Inst. For. Zvolensis.

[35] Ellenberg H., Weber H.E., Dull R., Wirth V., Werner W., Paulissen D. (2001). Zeigerwerte von Pflanzen in Mitteleuropa, Scripta Geobotanica.

[36] Voser-Huber M.L. (1983). Studien an eingeburgerten arten der gattung solidago L. Dissertat. Botan..

[37] Botta-Dukát Z. (2016). Morphological plasticity in the rhizome system of Solidago gigantea (Asteraceae): Comparison of populations in a wet and a dry habitat. Acta Bot. Hung..

[38] Hestir E.L., Khanna S., Andrew M.E., Santos M.J., Viers J.H., Greenberg J.A., Rajapakse S.S., Ustin S.L. (2008). Identification of invasive vegetation using hyperspectral remote sensing in the California Delta ecosystem. Remote Sens. Environ..

[39] Breiman L. (2001). Random forests. Mach. Learn..

[40] Cutler D.R., Edwards T.C., Beard K.H., Cutler A., Hess K.T., Gibson J., Lawler J. (2007). Random forests for classification in ecology. Ecology.

[41] Lawrence R.L., Wood S.D., Sheley R.L. (2006). Mapping invasive plants using hyperspectral imagery and Breiman Cutler classifications (randomForest). Remote Sens. Environ..

[42] Congalton R.G., Green K. (1999). Assessing the Accuracy of Remotely Sensed Data: Principles and Practices.

[43] Lillesand T., Kiedfer R., Chipman J. (2008). Remote Sensing and Image Interpretation.

[44] Vegetation Classification Studio Software, Version 2.13/hb. http://www.definity.pl/vcs.

[45] Ustin S.L., DiPietro D., Olmstead K., Underwood E., Scheer G.J. (2002). Hyperspectral remote sensing for invasive species detection and mapping. IEEE Int. Geosci. Remote Sens. Symp..

[46] Dorigo W., Lucieer A., Podobnikar T., Carni A. (2012). Mapping invasive Fallopia japonica by combined spectral, spatial, and temporal analysis of digital orthophotos. Int. J. Appl. Earth Obs. Geoinf..

[47] Mirik M., Ansley R.J., Steddom K., Jones D.C., Rush C.M., Michels G.J., Elliott N.C. (2013). Remote distinction of a noxious weed (Musk Thistle: Carduus Nutans) using airborne hyperspectral imagery and the support vector machine classifier. Remote Sens..

[48] Underwood E., Ustin S., Dipietro D. (2003). Mapping Non-Native Plants Using Hyperspectral Imagery. Remote Sens. Environ..

[49] Ishii J., Washitani I. (2013). Early detection of the invasive alien plant Solidago altissima in moist tall grassland using hyperspectral imagery. Int. J. Remote Sens..

[50] Evangelista P.H., Stohlgren T.J., Morisette J.T., Kumar S. (2009). Mapping invasive tamarisk (Tamarix): A comparison of single-scene and time-series analyses of remotely sensed data. Remote Sens..

[51] Vilà M., Schaffner U., Pyšek P., Pergl J., Jarošík V., Hulme P.E., Hejda M. (2011). A global assessment of invasive plant impacts on resident species, communities and ecosystems: The interaction of impact measures, invading species’ traits and environment. Glob. Chang. Biol..

[52] Millard K., Richardson M. (2013). Wetland mapping with LiDAR derivatives, SAR polarimetric decompositions, and LiDAR-SAR fusion using a random forest classifier. Can. J. Remote Sens..

[53] Peerbhay K., Mutanga O., Lottering R., Bangamwabo V., Ismail R. (2016). Detecting bugweed (Solanum mauritianum) abundance in plantation forestry using multisource remote sensing. ISPRS J. Photogramm. Remote Sens..

[54] Barbosa J.M., Asner G.P., Martin R.E., Baldeck C.A., Hughes F., Johnson T. (2016). Determining subcanopy Psidium cattleianum invasion in Hawaiian forests using imaging spectroscopy. Remote Sens..

[55] De Castro A.I., Jurado-Expósito M., Peña-Barragán J.M., López-Granados F. (2012). Airborne multi-spectral imagery for mapping cruciferous weeds in cereal and legume crops. Precis. Agric..

[56] Raczko E., Zagajewski B. (2017). Comparison of support vector machine, random forest and neural network classifiers for tree species classification on airborne hyperspectral APEX images. Eur. J. Remote Sens..

[57] Marcinkowska-Ochtyra A., Jarocińska A., Bzdęga K., Tokarska-Guzik B. (2018). Classification of expansive grassland species in different growth stages based on hyperspectral and LiDAR data. Remote Sens..

[58] Mundt J.T., Glenn N.F., Weber K.T., Prather T.S., Lass L.W., Pettingill J. (2005). Discrimination of hoary cress and determination of its detection limits via hyperspectral image processing and accuracy assessment techniques. Remote Sens. Environ..

[59] Schmidt J., Fassnacht F.E., Förster M., Schmidtlein S. (2017). Synergetic use of Sentinel-1 and Sentinel-2 for assessments of heathland conservation status. Remote Sens. Ecol. Conserv..

[60] Chen C., Liaw A., Breiman L. (2004). Using Random Forest to Learn Imbalanced Data.

[61] He K.S., Rocchini D., Neteler M., Nagendra H. (2011). Benefits of hyperspectral remote sensing for tracking plant invasions. Divers. Distrib..

