# Supplementary figures and images for: Using Airborne Hyperspectral Imaging Spectroscopy to Accurately Monitor Invasive and Expansive Herb Plants: Limitations and Requirements of the Method
Source: Sensors (Basel). 2019 Jun 28;19(13):2871. doi: 10.3390/s19132871 (PMC6651360; doi:10.3390/s19132871)

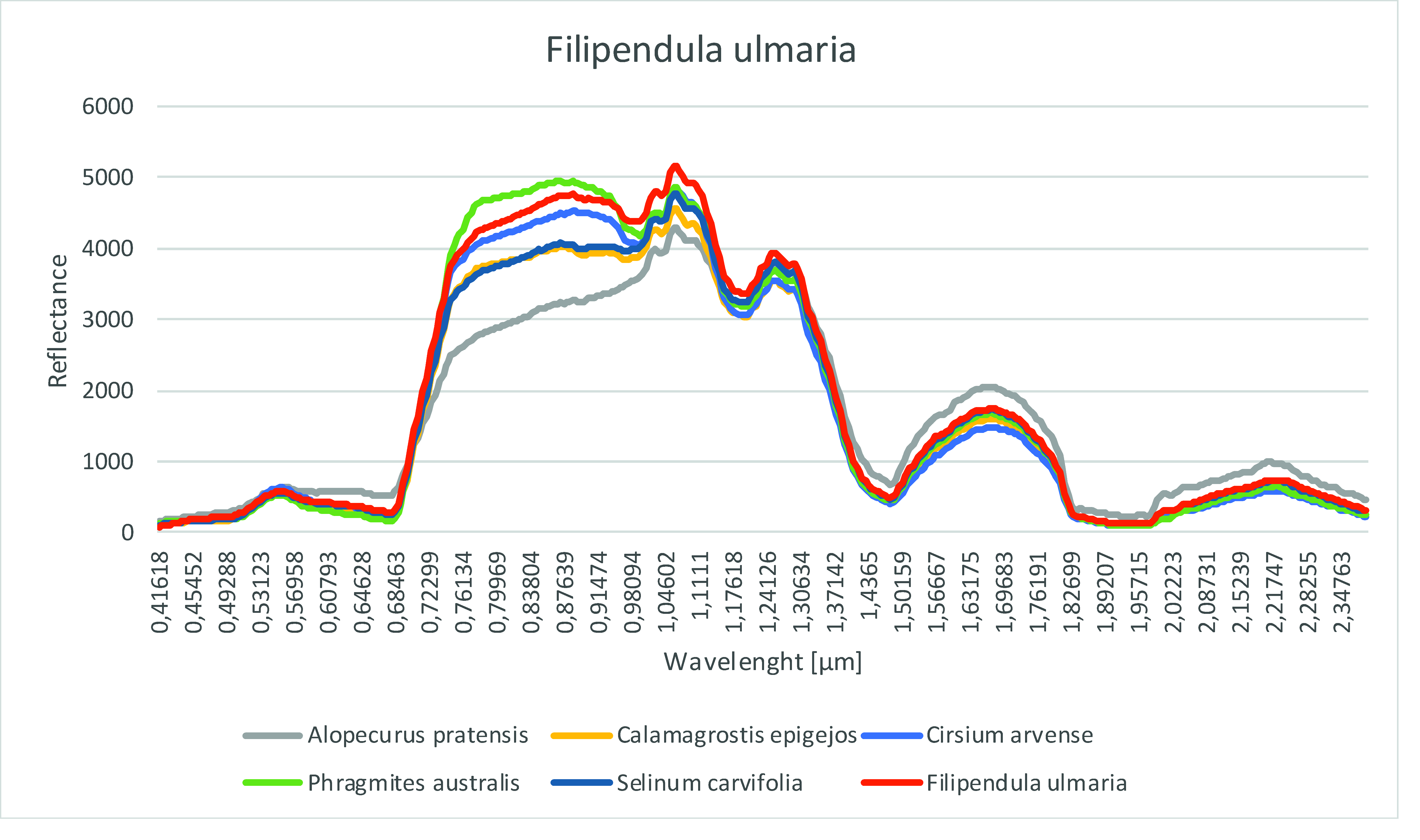

Supplement: Supplementary file 1 [file sensors-19-02871-s001.zip › Supplementary_materials/Spectral_profiles/FU_spectral_profile.jpg]

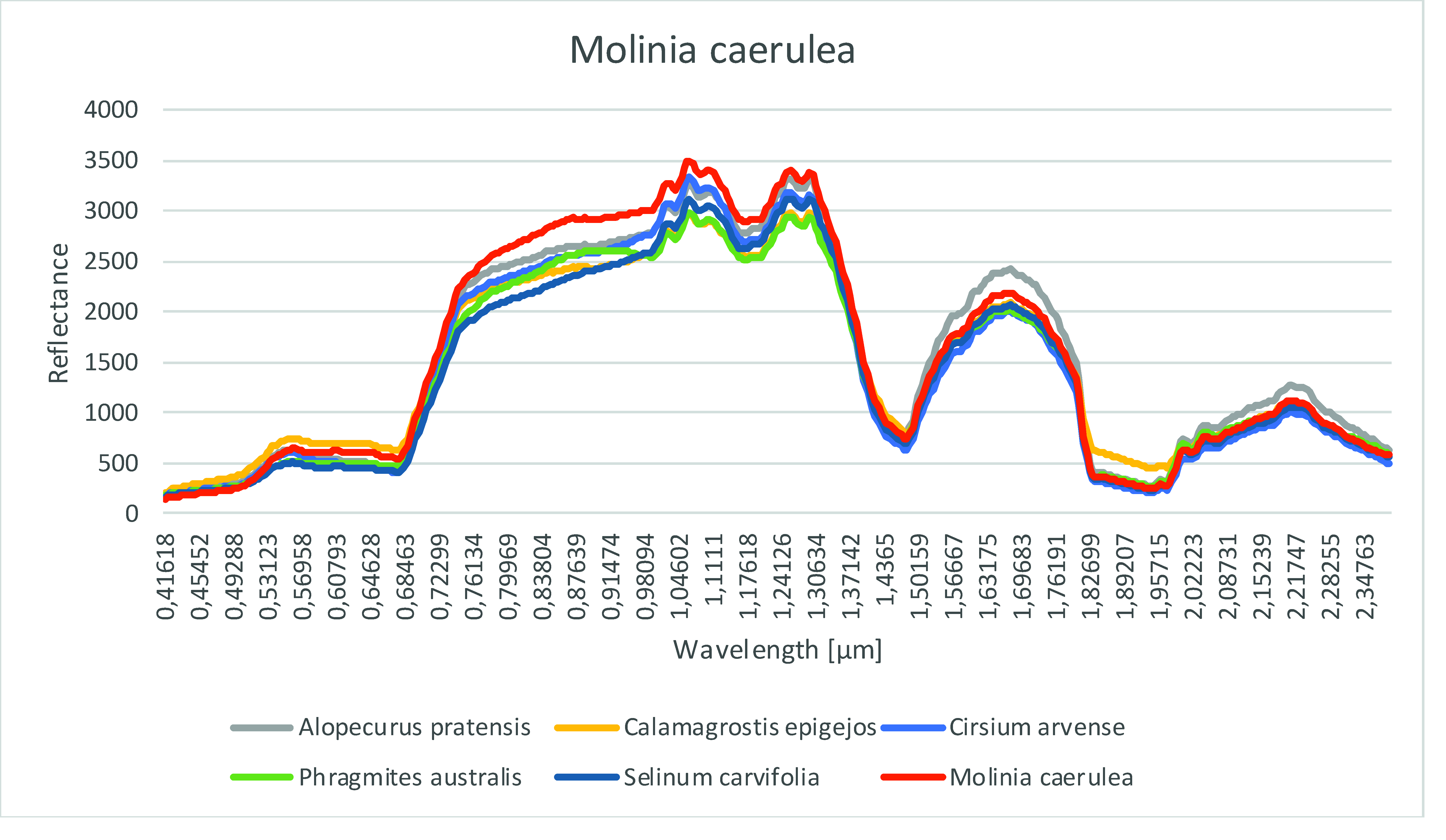

Supplement: Supplementary file 1 [file sensors-19-02871-s001.zip › Supplementary_materials/Spectral_profiles/MC_spectral_profile.jpg]

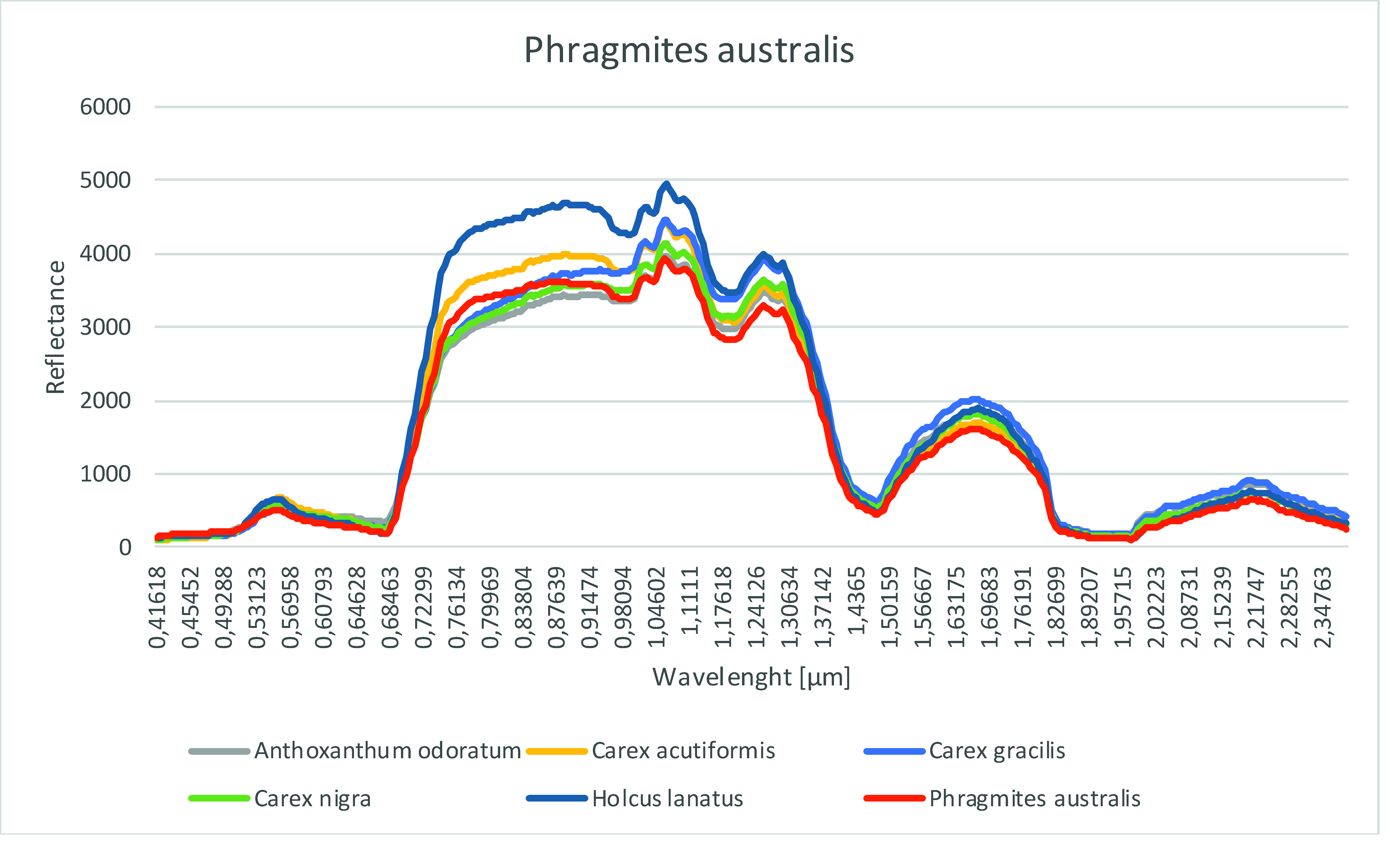

Supplement: Supplementary file 1 [file sensors-19-02871-s001.zip › Supplementary_materials/Spectral_profiles/PA_spectral_profile.jpg]

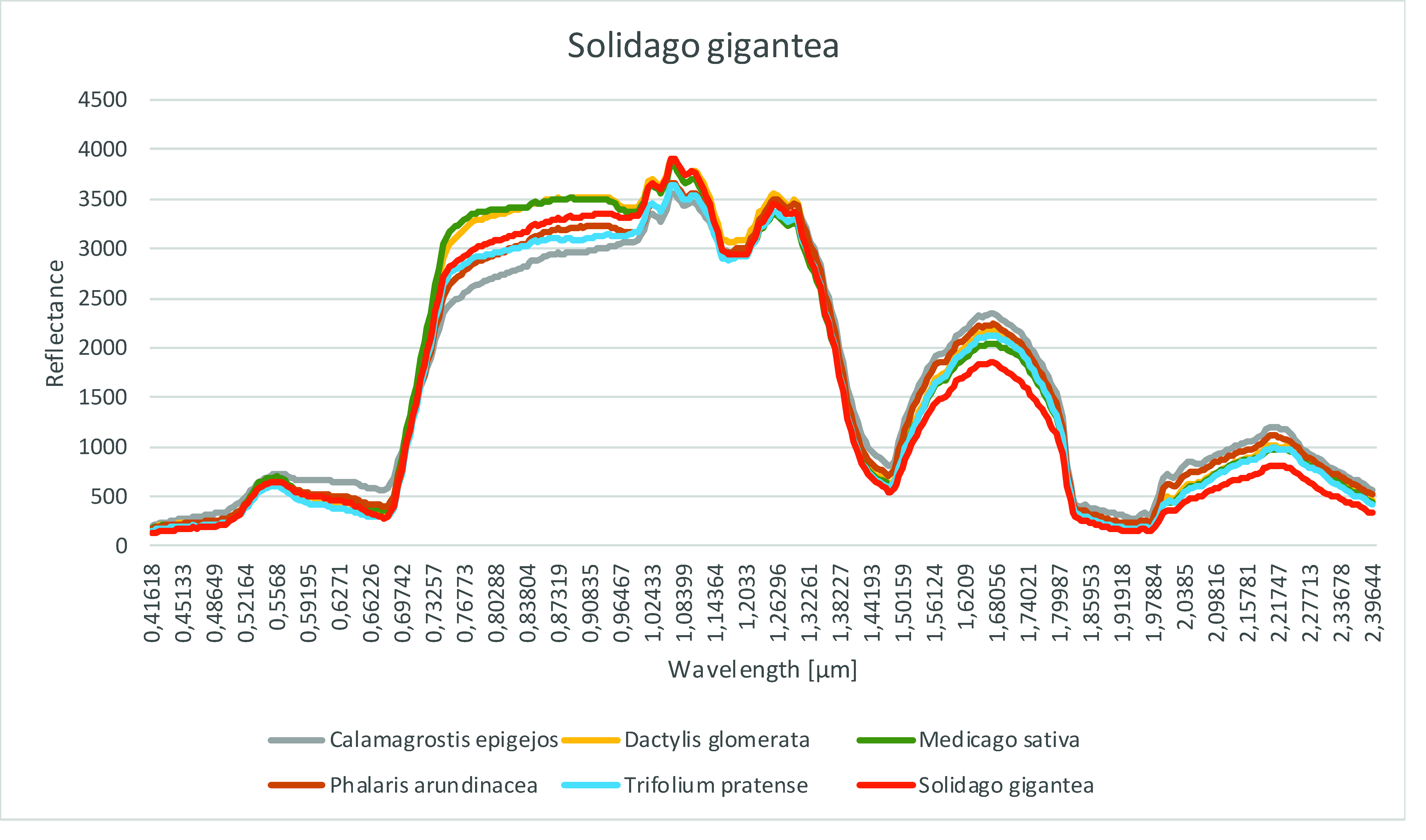

Supplement: Supplementary file 1 [file sensors-19-02871-s001.zip › Supplementary_materials/Spectral_profiles/SG_spectral_profile.jpg]

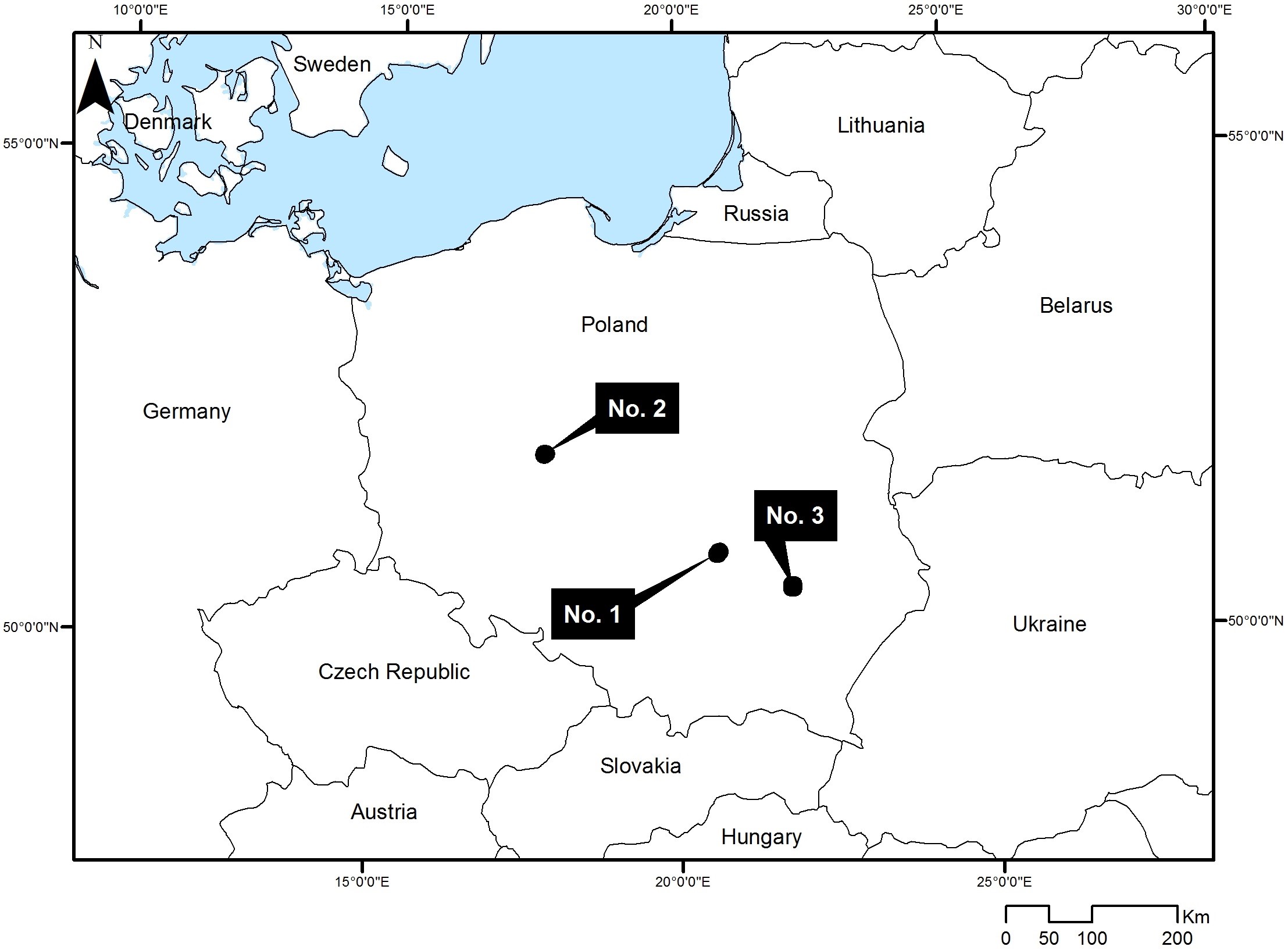

Supplement: Supplementary file 1 [file sensors-19-02871-s001.zip › Supplementary_materials/Study_areas/Study_areas.jpg]
